# Color liquid crystal grating based color holographic 3D display system with large viewing angle

**DOI:** 10.1038/s41377-023-01375-0

**Published:** 2024-01-15

**Authors:** Di Wang, Yi-Long Li, Fan Chu, Nan-Nan Li, Zhao-Song Li, Sin-Doo Lee, Zhong-Quan Nie, Chao Liu, Qiong-Hua Wang

**Affiliations:** 1 School of Instrumentation and Optoelectronic Engineering, Beihang University, Beijing, 100191 China; 2 State Key Laboratory of Virtual Reality Technology and Systems, Beihang University, Beijing, 100191 China; 3 Display Technology Research Center, Seoul National University, Gwanak-ro 1, Gwanak-gu, Seoul, 08826 Republic of Korea; 4 Key Lab of Advanced Transducers and Intelligent Control System, Ministry of Education, Taiyuan University of Technology, Taiyuan, 030024 China

**Keywords:** Displays, Liquid crystals

## Abstract

Holographic 3D display is highly desirable for numerous applications ranging from medical treatments to military affairs. However, it is challenging to simultaneously achieve large viewing angle and high-fidelity color reconstruction due to the intractable constraints of existing technology. Here, we conceptually propose and experimentally demonstrate a simple and feasible pathway of using a well-designed color liquid crystal grating to overcome the inevitable chromatic aberration and enlarge the holographic viewing angle, thus enabling large-viewing-angle and color holographic 3D display. The use of color liquid crystal grating allows performing secondary diffraction modulation on red, green and blue reproduced images simultaneously and extending the viewing angle in the holographic 3D display system. In principle, a chromatic aberration-free hologram generation mechanism in combination with the color liquid crystal grating is proposed to pave the way for on such a superior holographic 3D display. The proposed system shows a color viewing angle of ~50.12°, which is about 7 times that of the traditional system with a single spatial light modulator. This work presents a paradigm for achieving desirable holographic 3D display, and is expected to provide a new way for the wide application of holographic display.

## Abstract (web summary)

A color liquid crystal grating based color holographic 3D display system is proposed with large viewing angle.

## Graphical abstract

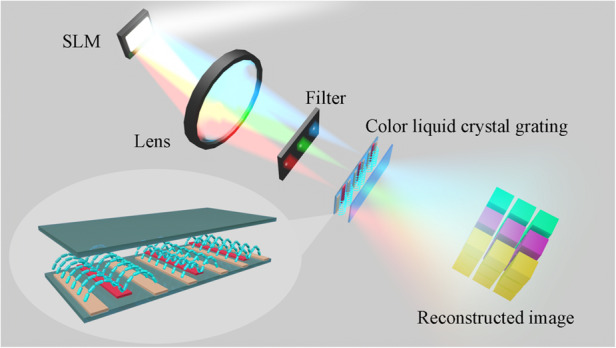

## Introduction

Holographic display technology can completely record and reconstruct the wavefront information of 3D objects, and it is one of the most promising naked-eye 3D display technologies^1,2^. Color holographic 3D display with large viewing angle has always been pursued, and it has important application values in medical treatment, industrial inspection, education and entertainment^3–5^. However, the color and viewing angle of holographic 3D display mainly depend on the wavelength of laser and the pixel size of current spatial light modulator (SLM). Inevitable color differences and narrow viewing angle seriously affect the holographic display effect and hinder the application of holographic 3D display in many fields.

In order to enlarge the viewing angle of holographic 3D display, many methods have been proposed^6–9^. For example, the method based on time or space multiplexing can enlarge the holographic viewing angle or realize full color by increasing the space bandwidth product of the system^10–15^. However, under the limited space bandwidth product, there lies a trade-off between color and large viewing angle. In addition, holographic optical element is also used in holographic systems to expand the viewing angle^16–19^. However, due to the wavelength selectivity of the holographic optical element, its application in color holographic display is very difficult. Besides, new optical modulation elements are used in holographic systems^20–23^. In 2017, researchers used scattering film and wavefront shaper to break through the limitation of SLM and achieved a viewing angle of 36°, but only monochrome holographic display was achieved^24^. In 2020, researchers used a coherent backlight unit to achieve a 30° color holographic display effect^25^.

Liquid crystal is a structured dynamic soft material that can be controlled by various external stimuli and presents several different textures based on its geometric constraints and applied external stimuli^26–35^. In order to eliminate the dispersion of liquid crystal diffraction devices, the Pancharatnam-Berry optical elements with specifically designed spectral response are proposed^1,36^. In 2022, we proposed a monochromatic holographic 3D display system based on liquid crystal grating, and a viewing angle of 57.4° was realized^37^. The basic optical mechanism of liquid crystal grating is to generate periodic electric field distribution in liquid crystal^38^. However, the diffraction angle of liquid crystal grating depends on the wavelength, which leads to the inevitable chromatic aberration problem and the application of liquid crystal grating in color display has also been seriously hindered. In recent years, although there have been many methods to expand the viewing angle of monochromatic holographic 3D display, color holographic 3D display technology with large viewing angle has not achieved a breakthrough, which limits the application of holographic display.

**Fig. 1:**
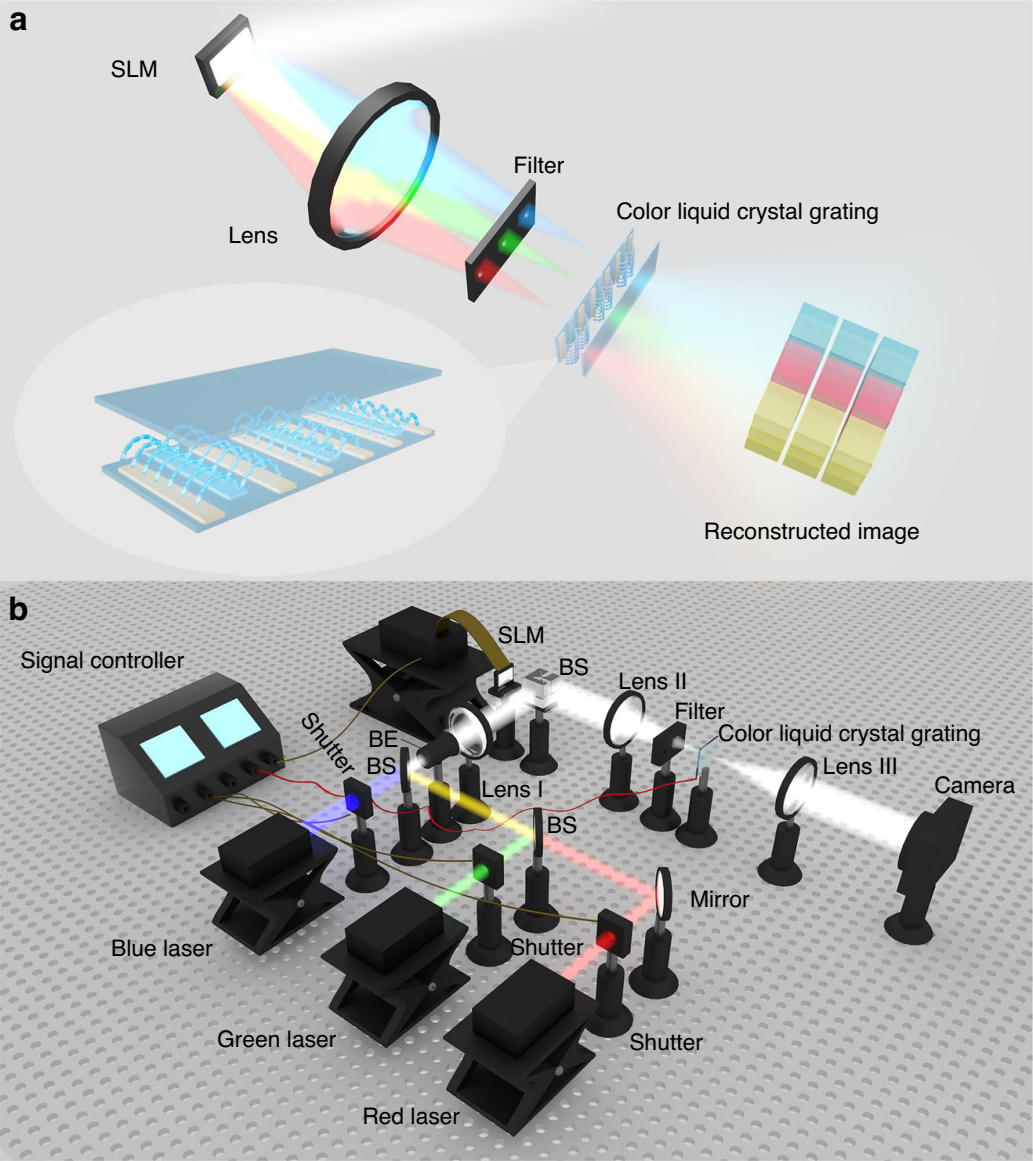
Concept and structure of the proposed system. **a** Concept of the proposed system. **b** Structure of the proposed system

Here, a color liquid crystal grating based holographic 3D display system with large viewing angle is proposed, as shown in Fig. 1a. Different from the traditional liquid crystal grating that has inevitable chromatic aberration, the proposed color liquid crystal grating can perform secondary diffraction modulation on red, green and blue reconstructed images with the same diffraction angle when the voltage is applied, thus avoiding chromatic aberration. In addition, a chromatic aberration-free hologram generation mechanism is proposed to cooperate with color liquid crystal grating to achieve large viewing angle color display. The proposed system shows a color viewing angle of ~50.12°, without any chromatic aberration. When the proposed system displays a monochrome holographic 3D image, the viewing angle can reach ~91.5°. The proposed system solves the problems of small viewing angle and serious chromatic aberration in the traditional holographic 3D display system, which has a decent display effect and broad application prospect.

## Results

### Structure of the proposed system

As shown in Fig. 1b, the proposed system consists of a red laser, a green laser, a blue laser, three shutters, three beam splitters (BSs), a mirror, a signal controller, a beam expander (BE), three lenses, an SLM, a filter and a color liquid crystal grating. Among them, the red laser, green laser, blue laser and three shutters are used to generate red, green and blue light emitted in time sequence. The mirror and two BSs are used to coincide the optical axes of red, green and blue light. The red, green and blue light with overlapping optical axes are reflected to the SLM by BS after passing through the BE and lens I. The corresponding blazed gratings are superimposed on the holograms. The loading order of red, green and blue holograms is consistent with the emergent order of red, green and blue light. The signal controller is a synchronization control system based on a programmable microcontroller. It is used to control the switching time of three shutters and three color holograms, and to control the voltage applied to the color liquid crystal grating. The color liquid crystal grating is located at the back focal plane of lens II and at the front focal plane of lens III. The holographic diffraction images of red, green and blue channels are incident on different liquid crystal layer regions of the color liquid crystal grating. The holographic diffraction images of three colors are modulated by secondary diffraction in different liquid crystal layers of the color liquid crystal grating, and the color diffraction images with identical intervals are reconstructed. Finally, the color holographic reconstructed image with large viewing angle can be received by the camera.

### Design of the color liquid crystal grating

**Fig. 2:**
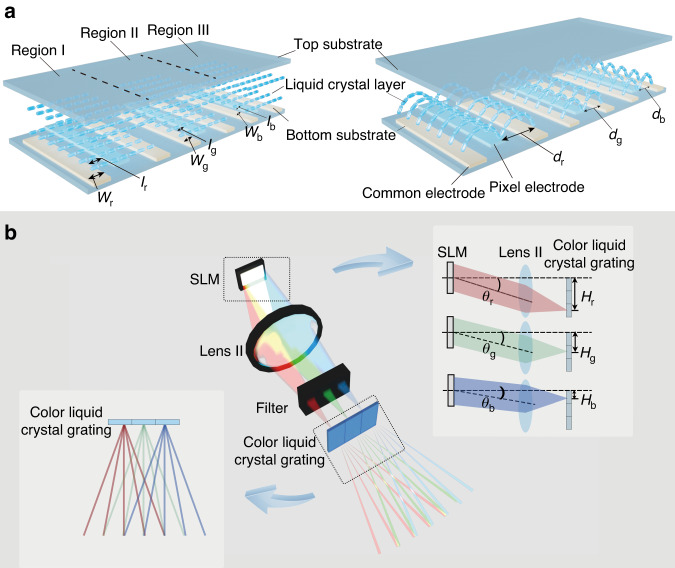
Realization of large viewing angle color holographic display. **a** Structure of the color liquid crystal grating. **b** Principle of the large viewing angle color holographic display

In order to make the three colors of light passing through the color liquid crystal grating have the same diffraction angle, we design and fabricate a liquid crystal grating with three different-pitch regions in one liquid crystal cell for different incident wavelengths, namely region I, region II and region III, as shown in Fig. 2a. The three regions of the color liquid crystal grating are controlled by the same voltage. The color liquid crystal grating consists of a top substrate, liquid crystal layer, pixel electrodes, common electrodes and a bottom substrate. The pixel and common electrodes are etched by planar indium tin oxide electrode. The common electrode widths of the three regions are *w*_r_, *w*_g_ and *w*_b_, respectively. In each region, the width of the pixel electrode is the same as that of the common electrode. The electrode arrangement is optimized by using a separate periodic electrode arrangement to eliminate the fringe electric field and diffraction crosstalk among the three regions. The pitches of the color liquid crystal grating in region I, region II and region III are *d*_r_, *d*_g_ and *d*_b_, respectively. The gaps between the common electrode and pixel electrode in region I, region II and region III are *l*_r_, *l*_g_ and *l*_b_, respectively. The same voltage is applied to the pixel electrodes in three regions, and the common electrodes in all regions are grounded. With this design, the arrangement periods of liquid crystal molecules in regions I, II and III of the color liquid crystal grating are different from each other when the voltage is applied, so the pitches of different regions are different accordingly. The pitch of region I is the largest, followed by region II and region III is the smallest. In addition, the adjacent electrodes between different regions are all set as common electrodes, thus the fringe electric field of adjacent regions can be eliminated and the angle disturbance of the liquid crystal molecules in adjacent regions decreased by 80%. Therefore, the control accuracy of different regions of the color liquid crystal grating can be improved.

### Chromatic aberration-free hologram generation mechanism for color holographic 3D display with large viewing angle

The generation mechanism of a hologram without chromatic aberration is as follows. Firstly, the information of red, green and blue color channels of a 3D object is extracted. Then, the signal controller is used to generate the hologram of each color channel, and the corresponding blazed grating is superimposed on the hologram. The resolution of hologram and blazed grating is the same as that of SLM, which is *a* × *b*. Where *a* is the lateral resolution and *b* is the vertical resolution. The parameters of blazed grating satisfy the following equations:

1 $$2d\,\sin \gamma =\lambda$$

2 $$d=np$$

where *d* is the pitch of the blazed grating, *γ* is the blazed angle of the blazed grating, *λ* is the wavelength, *p* is the pixel interval of the SLM, and *n* is the number of pixels in a single period of the blazed grating. *γ* is expressed by Eq. (3):

3 $$\gamma =\arctan \left(\frac{\varphi }{2\pi }\times \frac{\lambda }{d}\right)$$

where *φ* is the phase change of the blazed grating in each period, and 0 < *φ* ≤ 2π. When the blazed grating is superimposed on the hologram and the collimated light irradiates the hologram, the diffracted light field of the hologram has a certain deflection angle *θ* = 2*γ*.

The red channel hologram and blazed grating I, the green channel hologram and blazed grating II, and the blue channel hologram and blazed grating III are superimposed respectively, and then loaded on the SLM in time sequence. At time *T*_1_, only shutter I is opened. At this time, the SLM is irradiated by the red light, and the red hologram and blazed grating I are loaded on the SLM. At time *T*_2_, only shutter II is opened. At this time, the SLM is irradiated by the green light, and the green hologram and blazed grating II are loaded on the SLM. At time *T*_3_, only shutter III is opened. At this time, the SLM is irradiated by the blue light, and the blue hologram and blazed grating III are loaded on the SLM. According to the visual persistence effect of human eyes, three color reconstructed images can be seen at the same time. The holographic diffraction images are diffracted and modulated after passing through the color liquid crystal grating, and second-order diffraction images will be generated.

The wavelengths of the red light, green light and blue light are recorded as *λ*_r_, *λ*_g_ and *λ*_b_ respectively, and the holographic diffraction light fields of red, green and blue color channels respectively irradiate different liquid crystal layer regions of the color liquid crystal grating after passing through lens II. As shown in Fig. 2b, at time *T*_1_, the deflection angle of the holographic diffracted light field of the red channel is *θ*_r_. The holographic diffracted light field of the red channel passes through lens II with the focal length *f*, and converges to a point on the focal plane of lens II. Then, the light field is modulated by region I to generate *M* red second-order diffracted images. At this time, the interval *L*_r_ between two adjacent red second-order diffraction images and the distance *H*_r_ from the convergence point on the focal plane of lens II to the optical axis can be expressed as follows:

4 $${L}_{{\rm{r}}}=\frac{{\lambda }_{{\rm{r}}}f}{{d}_{{\rm{r}}}}$$

5 $${H}_{r}=f\,\tan {\theta }_{r}$$

At time *T*_2_, the deflection angle of the holographic diffracted light field of the green channel is *θ*_g_. The holographic diffracted light field of the green channel passes through lens II with the focal length *f*, and converges to a point on the focal plane of lens II. Then, the light field is modulated by region II to generate *M* green second-order diffracted images. At this time, the interval *L*_g_ between two adjacent green second-order diffraction images and the distance *H*_g_ from the convergence point on the focal plane of lens II to the optical axis can be expressed as follows:

6 $${L}_{{\rm{g}}}=\frac{{\lambda }_{{\rm{g}}}f}{{d}_{{\rm{g}}}}$$

7 $${H}_{g}=f\,\tan {\theta }_{g}$$

At time *T*_3_, the deflection angle of the holographic diffracted light field of the blue channel is *θ*_b_. The holographic diffracted light field of the blue channel passes through lens II with the focal length *f*, and converges to a point on the focal plane of lens II. Then, the light field is modulated by region III to generate *M* blue second-order diffracted images. At this time, the interval *L*_b_ between two adjacent blue second-order diffraction images and the distance *H*_b_ from the convergence point on the focal plane of lens II to the optical axis can be expressed as follows:

8 $${L}_{{\rm{b}}}=\frac{{\lambda }_{{\rm{b}}}f}{{d}_{{\rm{b}}}}$$

9 $${H}_{b}=f\,\tan {\theta }_{b}$$

We adjust the deflection angles *θ*_r_, *θ*_g_ and *θ*_b_ of the holographic diffraction light fields for the red, green and blue color channels. When *H*_r_-*H*_g_ = *L*_r_, *H*_g_-*H*_b_ = *L*_g_, and *H*_r_-*H*_b_ = 2*L*_b_, (*M*-2) color second-order diffraction images can be received by the camera. At this time, the viewing angle of the color holographic reconstructed image is (*M*-2)*β*, where *β* is color holographic 3D display angle when the color liquid crystal grating is in the voltage-off state. In order to obtain crosstalk-free color reconstructed images, the width of region II should be <2*L*_g_.

### Reconstruction process

In the experimental system, the wavelengths of the red, green and blue lasers are 638 nm, 520 nm and 450 nm, respectively. The SLM is a reflective phase-only SLM and its pixel size is 3.6 μm. The resolution and the refresh rate are 3840 × 2160 and 180 Hz, respectively. The phase modulation capability of the SLM can reach to 2π. The focal lengths of lens I, lens II and lens III are all 40 cm. The signal controller synchronously controls the on/off state of the three shutters and the generation and loading of holograms on the SLM.

The signal controller is used to generate the holograms of red, green and blue channels and the corresponding blazed gratings (Supplementary Material S1). The holograms and blazed gratings are loaded on the SLM in time sequence. The resolutions of the holograms and blazed gratings are both 3840 × 2160.

### Simulation and experiments of the color liquid crystal grating

**Fig. 3:**
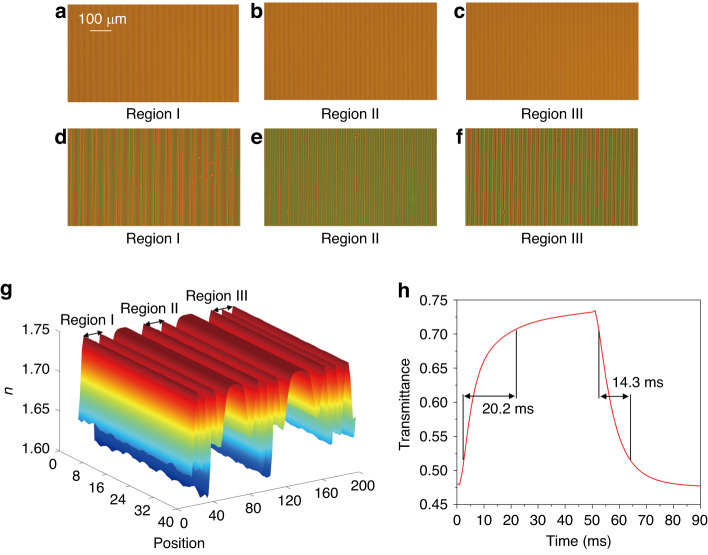
Test of the color liquid crystal grating. **a**–**c** Electrode structure and arrangement distribution in different regions of the color liquid crystal grating under the microscope in the voltage-off state. **d**–**f** Color liquid crystal grating distribution with different pitches under a polarization microscope in the voltage-on state. **g** Simulated effective refractive index distributions. **h** Response time of the color liquid crystal grating

In the color liquid crystal grating, the electrode widths of regions I, II and III are 13 μm, 10 μm and 9 μm respectively. The pitches of regions I, II and III are 26 μm, 20 μm and 18 μm respectively. Figure 3a–c show the electrode structure and arrangement distribution in different regions of the color liquid crystal grating under the microscope in the voltage-off state. When a voltage is applied to the pixel electrode, a spatially uneven gradient electric field distribution is formed between the pixel electrode and the common electrode, which induces the liquid crystal molecules to form a parabolic phase distribution, resulting in a light splitting effect similar to that of a phase grating. Figure 3d–f show the electrode structure and arrangement distribution in different regions of the color liquid crystal grating in the voltage-on state. As shown in Fig. 3d–f, in the voltage-on state, due to different pitches of each region of the liquid crystal grating, the three regions actually form three liquid crystal gratings with different periods. Besides, with the decrease of the pitch of the liquid crystal grating, the number of bright lines in the viewing area gradually increases accordingly. Moreover, the bright lines are evenly distributed and continuous.

Figure 3g shows the simulated effective refractive index distributions of the liquid crystal layer in different regions of the color liquid crystal grating. For ease of understanding, we simulated two periodic distributions for each region. As shown in Fig. 3g, in the voltage-on state, the effective refractive index distributions of the liquid crystal layer in the different regions are all axisymmetric, resulting in good diffraction effect. The periodic peak between adjacent grating regions is caused by the direction change of the liquid crystal molecules above the electrode. This periodical peak is very small and does not affect the holographic reconstructed results. The switching time is also a very important performance parameter of the color liquid crystal grating. The color liquid crystal grating is placed in the cross polarizers, and the switching time is tested by measuring the change of transmittance in the voltage on/off state. The rubbing direction of the liquid crystal molecules is set to 0°, and the directions of the polarizer and the analyzer are −45° and +45° respectively. As shown in Fig. 3h, in the voltage-on state, the time for the liquid crystal molecules to form the color liquid crystal grating is 20.2 ms. Due to the anchoring effect of the alignment layer, the recovery time after removing the voltage is 14.3 ms.

**Fig. 4:**
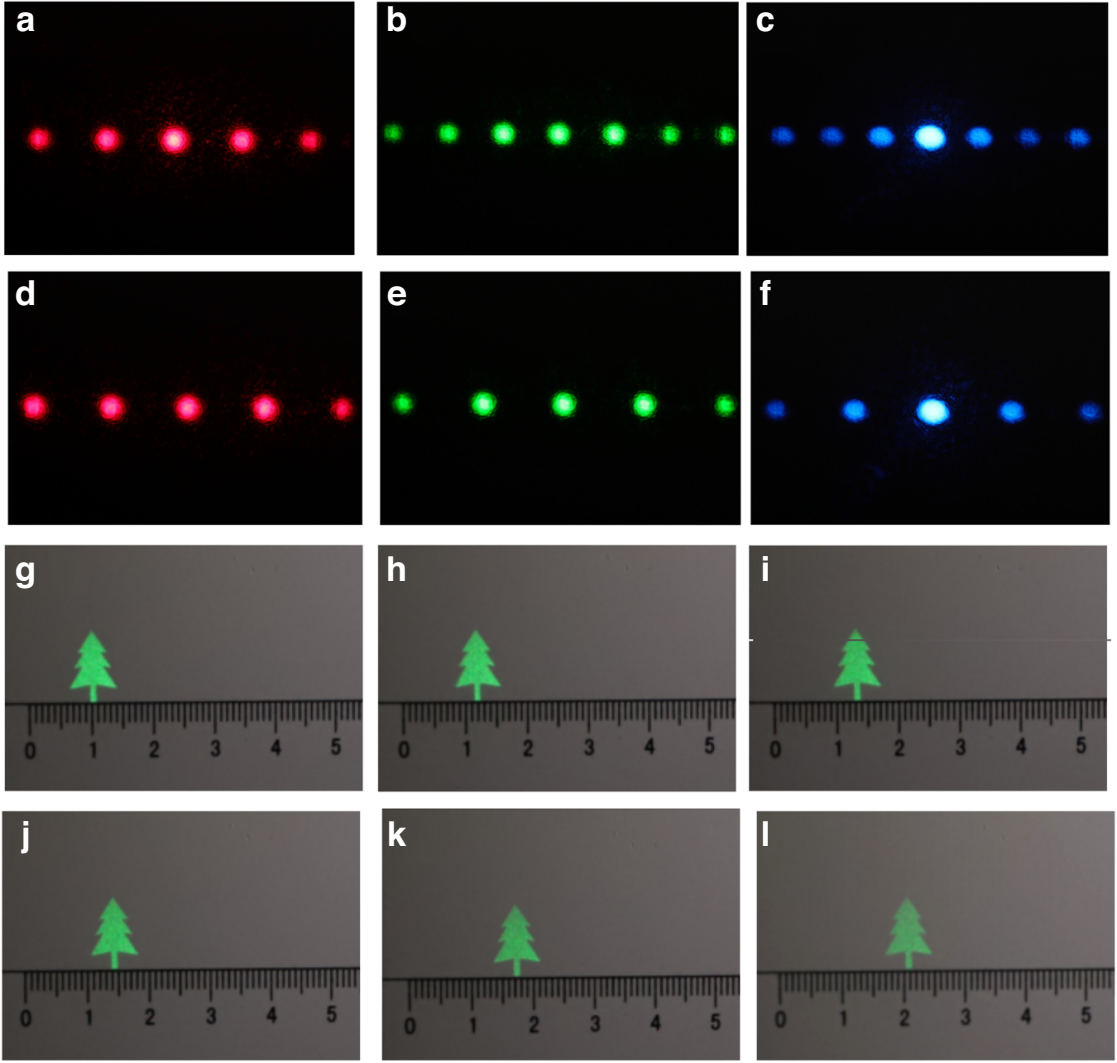
Optical experimental results of the color liquid crystal grating and blazed grating. **a**–**c** Distribution pattern when red, green and blue lasers irradiate the traditional liquid crystal gratings respectively. **d**–**f** Distribution pattern when red, green and blue lasers irradiate the proposed color liquid crystal gratings respectively. **g**–**l** Movement results of the reconstructed image when different blazed gratings are superimposed on the hologram

The distribution of diffracted light when red, green, and blue lasers irradiate the traditional liquid crystal gratings is shown in Fig. 4a–c. The traditional liquid crystal gratings have the same pitch. It can be seen that at this time, the distance of the adjacent diffraction orders between the red diffracted light is larger than that of the green diffracted light, while the distance between the blue diffracted light is the smallest. That is to say, when the traditional liquid crystal grating is used for secondary diffraction, chromatic aberration exists. Then, the designed color liquid crystal grating is used for diffraction, as shown in Fig. 4d–f. When the red, green and blue lasers respectively irradiate the proposed color liquid crystal grating, the distances between the red, green and blue diffracted lights are the same, and there is no chromatic aberration at this case (Supplementary Material S2).

### Experiment results of the proposed system with large viewing angle

The holographic diffraction light fields of three channels generated by the SLM pass through lens II and then enter the corresponding regions of the color liquid crystal grating. The holographic diffraction light fields of the three channels of red, green and blue are diffracted by the second order respectively, so that the interval between the second-order diffraction images of each color channel is 1 cm. In order to ensure that the spectral information of red, green and blue diffraction images can pass through the corresponding regions of color liquid crystal grating, we superimpose different blazed gratings on the holograms. The green reconstructed image (the resolutions of the object is 950 × 1350) is taken as an example, when blazed gratings with different blazed angles are superimposed on the hologram (Supplementary Material S3), the reconstructed image moves accordingly, as shown in Fig. 4g–l.

**Fig. 5:**
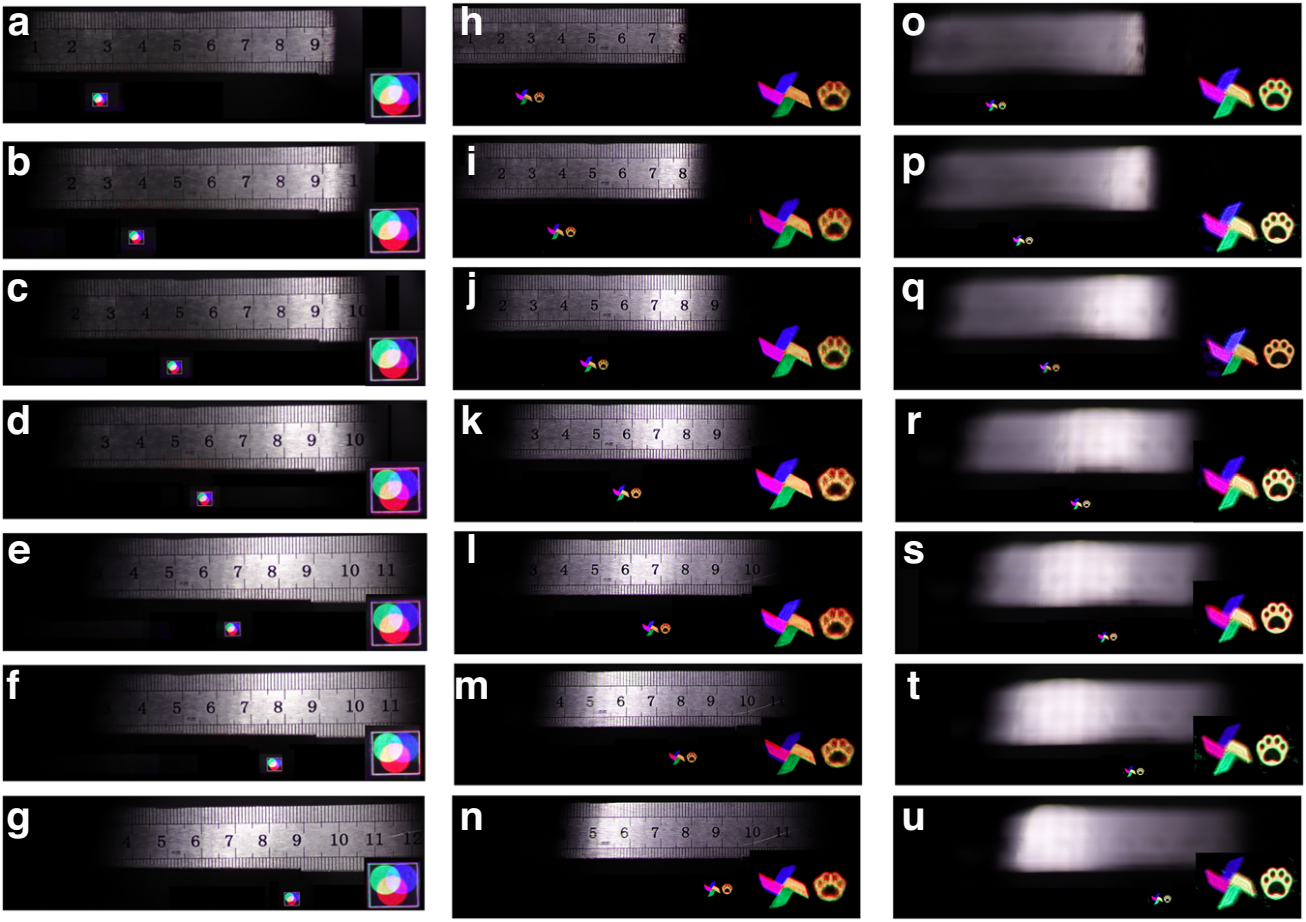
Color holographic reconstruction with large viewing angle. **a**–**g** Reconstructed image with different viewing areas of 2D object. **h-n** Reconstructed images with different viewing areas of 3D object when “windmill” is focused. **o**–**u** Reconstructed images with different viewing areas of 3D object when “cartoon bear paw” is focused

By controlling the incident angle of the light beam entering the color liquid crystal grating, the holographic diffracted light fields of the three channels of red, green and blue are modulated, respectively, and nine second-order diffracted images with equal spacing are generated. The diffraction order is influenced by the performance of the color liquid crystal grating itself. This has a high requirement for machining accuracy. In the experiment, we hope that the intensity of each second-order diffraction image is consistent. In the final experiment, nine uniform diffraction orders are realized. The maximum viewing angle is affected by the wavelength. The larger the wavelength is, the larger the viewing angle of the holographic display is. When only the red holographic 3D image is displayed, the viewing angle can reach ~91.5°, which is the largest viewing angle as far as we know. So, seven completely coincident color second-order diffracted images are obtained, and the large viewing angle color holographic 3D display can be realized. In color reconstruction, the viewing angle of the color reconstructed image is influenced by the blue wavelength. The real reference “straightedge” is placed on the same depth plane as the reconstructed image, as shown in Fig. 5a–g, and the resolution of the 2D color object is 1260 × 1150.

The camera moves horizontally from the leftmost side of the viewing plane to the right side. When the proposed system is used, nine secondary diffraction images of red, green and blue color channels appear. At this time, the viewing angle of the color holographic reconstructed image is ~50.12°. While the viewing angle of the color holographic reconstructed image without using the liquid crystal grating is only ~7.16°. In order to see the details of the reproduced image, an enlarged image of the reproduced image is placed in the lower right corner of each sub-image.

In addition, the color reproduction of 3D object is verified by experiment. The “windmill” and “cartoon bear paw” in different depths are used as the 3D object and their depths are 5 cm and 15 cm respectively. The resolution of the 3D object is 2100 × 1000. The real reference “straightedge” is used as a reference. When the reproduction distance is 5 cm, with the movement of the camera, the color holographic 3D display effect of focused “windmill” can be seen at different positions (Supplementary Material S4), and the reproduced image has no color difference, as shown in Fig. 5h–n. When the reproduction distance is 15 cm, with the movement of the camera, we can see the color 3D display effect of large viewing angle holography when “cartoon bear paw” is in focus, as shown in Fig. 5o-u. In the lower right corner of each sub-image is an enlarged view of the reproduced image. In the experiment, two depths are used for 3D demonstration. Theoretically, holographic 3D display technology can achieve continuous depth holographic reconstruction. However, because the camera has a certain depth of field, it is difficult to distinguish the positional relationship when the distance between objects in two adjacent depth planes is too small, and even there will be serious crosstalk.

**Fig. 6:**
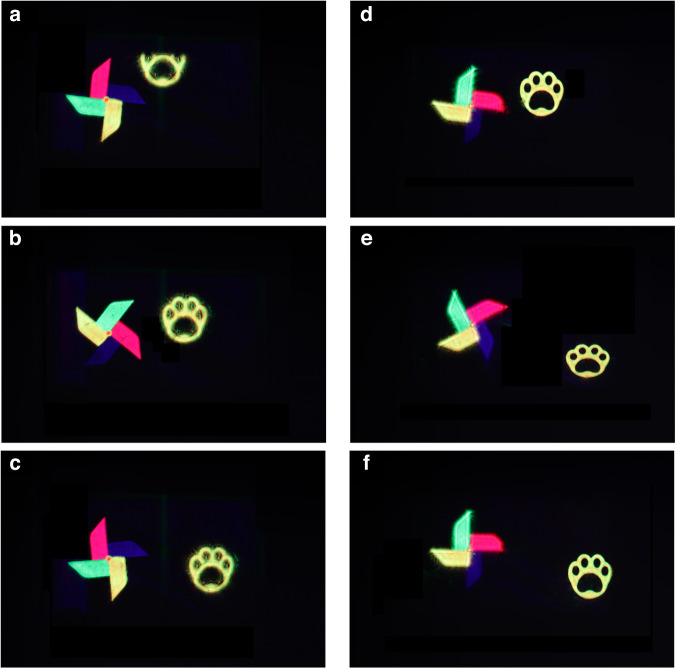
Reproduction results of the moving object. **a**–**c** Movement results of the reconstructed images when “windmill” is focused. **d**–**f** Movement results of the reconstructed images when “cartoon bear paw” is focused

In addition, the motion video of the 3D object is taken. The video of the “cartoon bear paw” from appearing to disappearing when the “windmill” is rotating is recorded (see Supplementary video 1 and Supplementary video 2). At different moments of the moving 3D object, the camera is used to capture the result when “windmill” is focused, as shown in Fig. 6a–c. Figure 6d–f shows the movement results at different moments when “cartoon bear paw” is focused. In the holographic reconstruction, the high coherence of laser will introduce speckle noise. Besides, the optical elements used in the holographic system may introduce additional phase difference, thus introducing noise. In addition, the random phase introduced by the hologram in the encoding calculation process will also cause speckle noise. There are many methods to suppress the speckle noise, including light source optimization method, iterative algorithm, deep learning algorithm and error diffusion method. In this paper, we optimize the phase distribution of the hologram, thus reducing the interference between adjacent pixels and effectively suppressing the noise of the reconstructed image. Speckle noise suppression is also an important direction in holographic 3D display, and we will continue to study it in the future. In the aspect of system integration, we can consider integrating RGB light source and replacing 4 *f* system with holographic optical elements, thus reducing the volume of the whole system.

## Discussion

We have first demonstrated an easy-to-implement method to realize color holographic 3D display system with large viewing angle. To this end, a specially structured color liquid crystal grating is designed on demand and a novel chromatic aberration-free hologram generation mechanism is put forward. In this case, chromatic aberration-free regulation of holographic reconstructed images with different wavelengths can be realized, thereby achieving large-viewing-angle and color holographic 3D display. The proposed holographic 3D display system enlarges the viewing angle of color holography to ~50.12°, which solves the problems of small viewing angle and serious chromatic aberration in the traditional holographic 3D display. The proposed mechanism can enrich the holographic 3D display theory. The proposed system has a simple structure and is expected to be applied in medical, industrial and other fields.

## Materials and methods

### Sample fabrication

In order to fabricate the color liquid crystal grating, firstly, a planar indium tin oxide electrode is deposited on the inner surface of the bottom substrate, and the planar indium tin oxide electrode of the bottom substrate is etched into three different periodic strip indium tin oxide electrodes. Secondly, the polyimide layer is coated on the inner surface of the bottom substrate and rubbed in the direction which is perpendicular to the periodic strip indium tin oxide electrodes. Thirdly, a top glass substrate without indium tin oxide electrode is coated with polyimide layer and rubbed in the same direction as the rubbing direction of the bottom substrate. Fourthly, the top glass substrate and the bottom substrate are encapsulated into a cell. Finally, the liquid crystal material is poured into the cell, and then the liquid crystal director distribution is homogeneously aligned perpendicular to the periodic strip indium tin oxide electrodes direction.

### Supplementary information

Supplemental Information for Color liquid crystal grating based color holographic 3D display system with large viewing angle

Supplementary video 1

Supplementary video 2
