# Supplementary material for: Color liquid crystal grating based color holographic 3D display system with large viewing angle
Source: Light Sci Appl. 2024 Jan 15;13:16. doi: 10.1038/s41377-023-01375-0 (PMC10788332; doi:10.1038/s41377-023-01375-0)
Supplement: Supplementary file 1 — Supplemental Information for Color liquid crystal grating based color holographic 3D display system with large viewing angle [file 41377_2023_1375_MOESM1_ESM.docx]

SUPPLEMENTARY MATERIAL

Color liquid crystal grating based color holographic 3D display system

with large viewing angle

Di Wang^1, 2, †^, Yi-Long Li^1,†^, Fan Chu^1, †^, Nan-Nan Li^1^, Zhao-Song Li^1^,

Sin-Doo Lee^3^, Zhong-Quan Nie^4^, Chao Liu^1^ and Qiong-Hua Wang^1, 2*^

1*School of Instrumentation and Optoelectronic Engineering, Beihang University, Beijing 100191, China.*

2*State Key Laboratory of Virtual Reality Technology and Systems, Beihang University, Beijing 100191, China*

3*Display Technology Research Center, Seoul National University, Gwanak-ro 1, Gwanak-gu, Seoul 08826, Republic of Korea.*

^4^ *Key Lab of Advanced Transducers and Intelligent Control System, Ministry of Education, Taiyuan University of Technology, Taiyuan 030024, China.*

^†^*These authors contributed equally to this work.*

**Correspondence*: *Qiong-Hua Wang, E-mail: [qionghua@buaa.edu.cn](mailto:qionghua@buaa.edu.cn)*

4 pages, 3 figures S1-S3

**S1: Change of spectral light field by using blazed grating**

The green light field is taken as an example to verify the change of the blazed grating on the light field of the spectrum position. A receiving screen is placed at the focal plane of lens II to observe the change of the light field at the spectral point. As shown in Fig. S1(a), the brightest point on the left is the zero-order light caused by the SLM, and the point on the right is the spectrum light spot. When different blazed gratings are superimposed on the hologram, the position of the zero-order light remains unchanged, while the position of the spectral light field changes, as shown in Figs. S1(b)-(e). With the change of blazed grating, the position of spectrum light field moves from the scale of 5.7 to 5.2. This also means that by superimposing the blazed grating on the hologram, the light fields of different colors can be controlled to pass through the corresponding areas of the color liquid crystal grating, thus realizing the secondary diffraction modulation of the reconstructed image of the corresponding color.


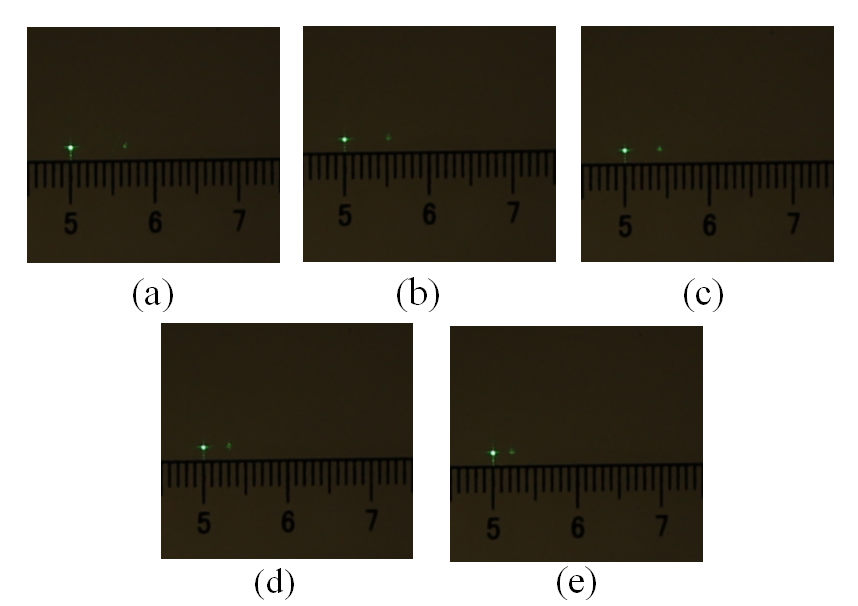


(e)

(d)

(c)

(b)

(a)

**Figure S1. a-e Position change of the spectrum point when different blazed gratings are superimposed on the hologram.**

**S2: Diffraction efficiency measurement of the color liquid crystal grating**

Fig. S2(a) shows the structure of the diffraction efficiency measurement system, which consists of a laser, a polarizer, a color liquid crystal grating and a photometer. The wavelengths of the red, green and blue lasers are 638 nm, 520 nm and 450 nm, respectively. The photometer used in the experiments is TES-1330A. The measurement error range is controlled within 0.01 cd. The red wavelength is tested as an example, Fig. S2(b) shows the diffraction efficiency of zero, first, second, third and fourth orders. The diffraction efficiencies of the 1st, 2nd, 3rd and 4th orders are similar at 7.5 V.


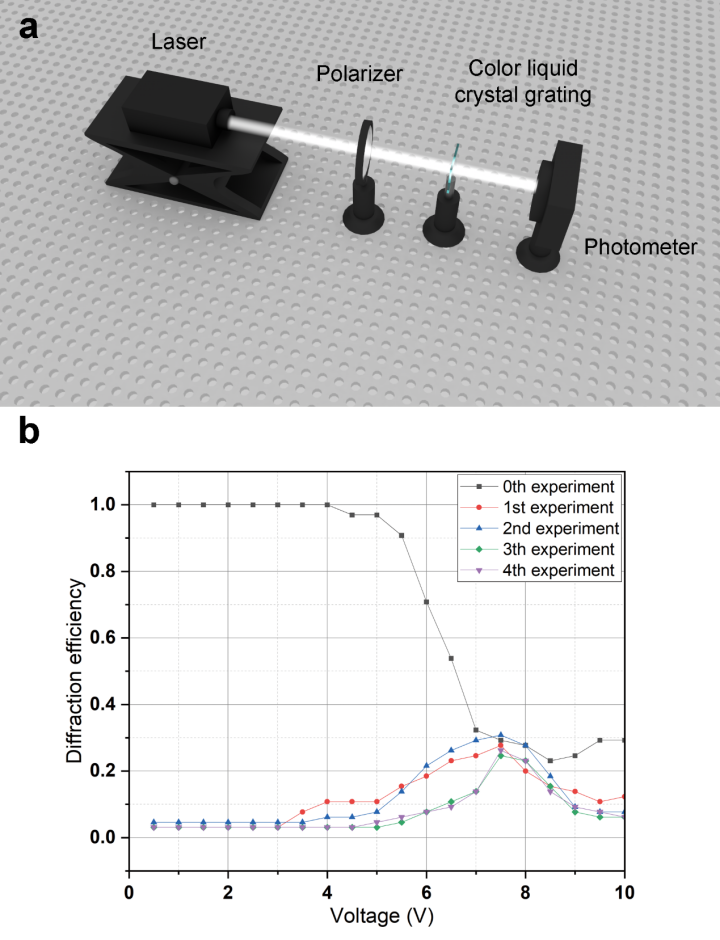


**Figure S2. Diffraction efficiency measurement of the color liquid crystal grating. a** Structure of the diffraction efficiency measurement system. **b** Result of the diffraction efficiency.

**S3: Calculation of the hologram**

In the holographic 3D display system, the optimized segmentation algorithm is designed based on the novel look-up-table (NLUT) algorithm to generate the hologram. The 3D object can be treated as a set of discretely sliced images. Each image at a fixed depth is approximated as a collection of emissive object points^1^. In this method, only the fringe patterns of the center points on each image plane are pre-calculated and stored in the memory, called principal fringe patterns (PFPs). Then, the fringe patterns for other object points on each image plane, called sub-computer generated holograms (sub-CGHs), can be obtained by simply shifting the pre-calculated PFPs according to the displaced values from the center to those points. The hologram of the 3D object can be generated by adding the sub-CGHs corresponding to all points.

Compared with the traditional point source model algorithm, the NLUT algorithm retains only simple addition operations, reduces the complexity of the calculation and therefore improves the calculation speed. However, when ultra-high-resolution SLM and 3D objects are used, the amount of calculation is greatly increased and the calculation time of the hologram becomes longer. Therefore, the improved NLUT algorithm is used to further improve the calculation speed^2^. In this method, the relationship between the pixels on the hologram and the corresponding reconstructed image is calculated firstly. Then, the sub-CGH corresponding to the object point of the recorded object is optimized and divided into two diffraction areas, the optimized diffraction area and the invalid diffraction area. After this, the optimized diffraction area of the sub-CGH for each object point is pre-calculated and saved. Finally, the final hologram can be generated by superimposing all the sub-CGHs. With the proposed method, the calculation time for the final hologram can be significantly reduced and the quality of the reconstructed image is not affected.

**S4:** **Large viewing reconstruction images** **with different color channels**

The large viewing reconstruction images of the “windmill” and “cartoon bear paw” with different color channels are shown in Fig. S3. Figs. S3(a)-(g) are the red reconstructed images with seven different viewing areas when “windmill” is focused, Figs. S3(h)-(n) are the green reconstructed images with seven different viewing areas when “windmill” is focused. Figs. S3(o)-(u) are the blue reconstructed images with seven different viewing areas when “windmill” is focused. It can be seen that the holographic reconstruction images of the RGB color channels are in the correct position.

**
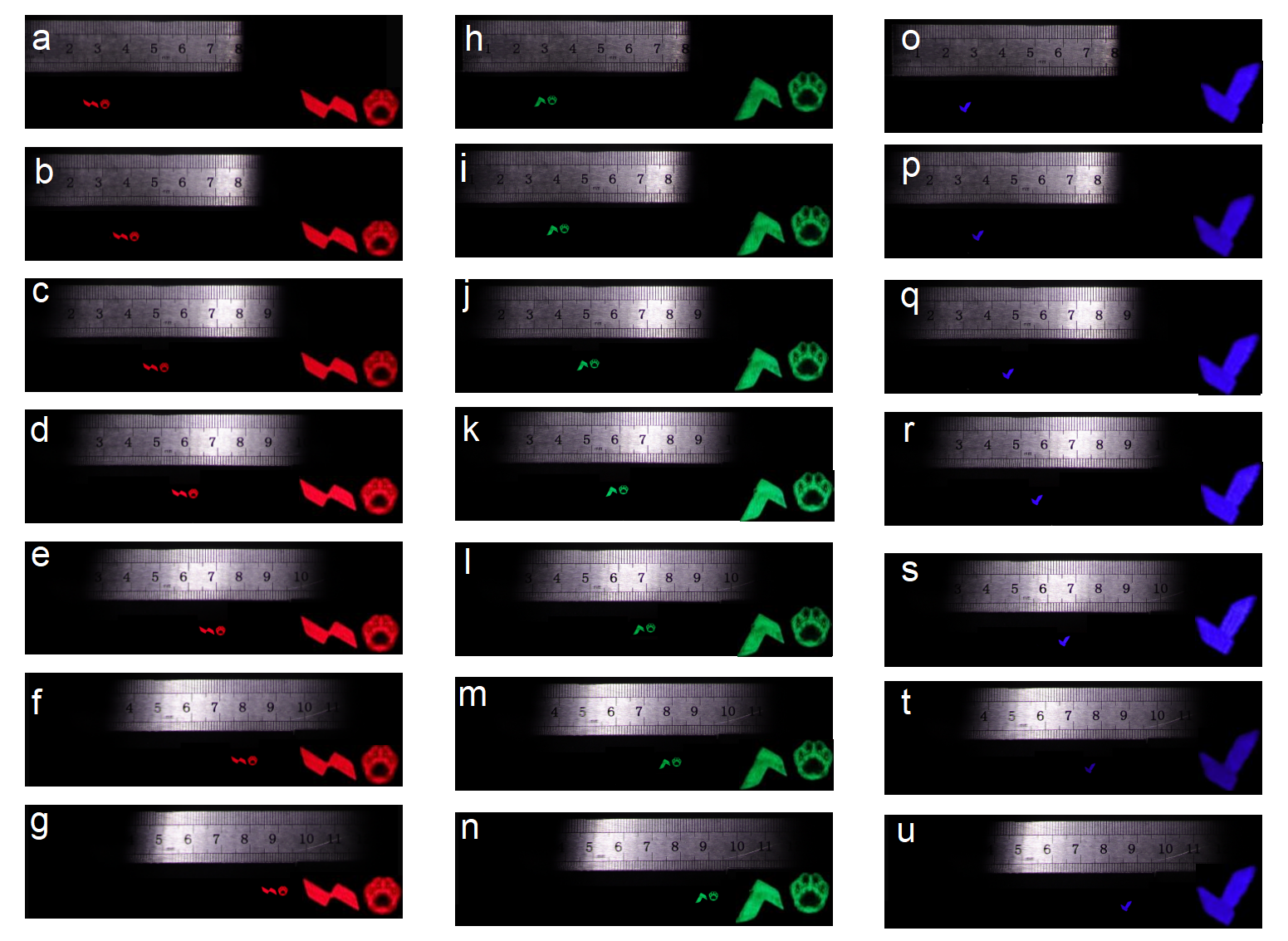
**

**Figure S3.** **Large viewing reconstruction** **images with different color channels. a-g** Red reconstructed images with seven different viewing areas when “windmill” is focused. **h-n** Green reconstructed images with seven different viewing areas when “windmill” is focused. **o-u** Blue reconstructed images with seven different viewing areas when “windmill” is focused.

**References**

1. Kim, S. C., & Kim, E. S. Effective generation of digital holograms of three-dimensional objects using a novel look-up table method. *Appl. Opt.* **47**(19), D55-D62 (2008).
2. Li, Y. L. *et al*. Fast hologram generation method based on optimal segmentation of sub-computer-generated hologram. *Opt. Express* **28**(21), 32185-32198 (2020).
